# Biosensor Technologies for Water Quality: Detection of Emerging Contaminants and Pathogens

**DOI:** 10.3390/bios15030189

**Published:** 2025-03-15

**Authors:** Antía Fdez-Sanromán, Nuria Bernárdez-Rodas, Emilio Rosales, Marta Pazos, Elisa González-Romero, Maria Ángeles Sanromán

**Affiliations:** 1 CINTECX, Universidade de Vigo, BIOSUV, Departamento de Ingeniería Química, 36310 Vigo, Spain; antia.fernandez.sanroman@uvigo.gal (A.F.-S.); nuria.bernardez@uvigo.gal (N.B.-R.); emiliorv@uvigo.es (E.R.); mcurras@uvigo.gal (M.P.); 2 Department of Analytical and Food Chemistry, Universidade de Vigo, Campus As Lagoas-Marcosende, 36310 Vigo, Spain; eromero@uvigo.es

**Keywords:** electrochemical biosensors, emerging contaminants, microfluidic system, optical biosensors, pathogens, piezoelectric biosensors, water quality assessment

## Abstract

This review explores the development, technological foundations, and applications of biosensor technologies across various fields, such as medicine for disease diagnosis and monitoring, and the food industry. However, the primary focus is on their use in detecting contaminants and pathogens, as well as in environmental monitoring for water quality assessment. The review classifies different types of biosensors based on their bioreceptor and transducer, highlighting how they are specifically designed for the detection of emerging contaminants (ECs) and pathogens in water. Key innovations in this technology are critically examined, including advanced techniques such as systematic evolution of ligands by exponential enrichment (SELEX), molecularly imprinted polymers (MIPs), and self-assembled monolayers (SAMs), which enable the fabrication of sensors with improved sensitivity and selectivity. Additionally, the integration of microfluidic systems into biosensors is analyzed, demonstrating significant enhancements in performance and detection speed. Through these advancements, this work emphasizes the fundamental role of biosensors as key tools for safeguarding public health and preserving environmental integrity.

## 1. Introduction

The global water crisis is critical, as climate change is causing severe meteorological alterations such as prolonged droughts and intense torrential rains. These events lead to a significant reduction in available water resources and a deterioration in their quality. The situation is further exacerbated by the increasing water consumption due to population growth, which has dramatically accelerated water scarcity [1]. Additionally, the limited water available is being contaminated by various chemical compounds, many of which are toxic and harmful to both human health and ecosystems.

Among these contaminants, emerging contaminants (ECs) stand out. ECs refer to synthetic and natural chemical agents, as well as biological agents, whose presence in the environment is not often monitored, but which have the potential to affect ecosystems and public health [2]. These contaminants often lack regulatory standards, and their environmental behavior and toxicological profiles are not fully understood [3]. ECs encompass a wide range of pollutants, including pharmaceuticals, micro- or nanoplastics, engineered nanoparticles, perfluoroalkylated and polyfluoroalkylated substances, pesticides, and industrial chemicals. These substances often resist natural degradation processes and require long-range transport, bioaccumulation, and complex ecosystem interactions, which complicates risk assessment [4].

**Figure 1:**
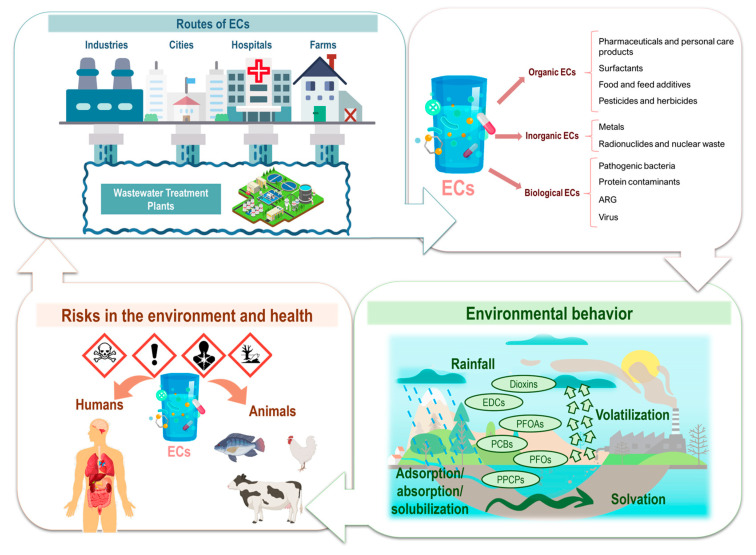
Release routes of emerging pollutants to the environment and eventual effects. EDCs: endocrine-disrupting chemicals; PFOAs: perfluooctanoic acids; PFOs: perfluorooctane sulfonates; PCBs: polychlorinated biphenyls; PPCPs: pharmaceuticals and personal care products.

The importance of studying ECs lies in their intensive use in various fields, such as pharmaceuticals, personal care products, agricultural pesticides, and industrial additives [2,5]. Furthermore, urban and industrial wastewater treatment plants are not efficient enough to eliminate these compounds, resulting in their reintroduction into the environment, where they persist and may accumulate [6]. A study by Fatta-Kassinos et al. [7] highlights that these compounds can persist in the environment and are being reintroduced into the human food chain through drinking water. The release routes of EC to the environment and eventual effects are disclosed in Figure 1. In traditional wastewater treatment plants, pharmaceuticals, disinfectants, and biological contaminants such as antibiotic-resistant bacteria and viruses are not effectively removed. As a result, these pollutants remain untreated, allowing them to enter the environment, where they can accumulate, spread, and pose significant risks to human health and ecosystems [8].

The presence of pharmaceutical contaminants in ecosystems not only disrupts aquatic life, but also fosters the development of antibiotic-resistant bacteria (ARB) and antibiotic resistance genes (ARGs) [9,10]. This situation arises because microorganisms exposed to sub-lethal concentrations of antibiotics in the environment can develop resistance mechanisms. Consequently, ARB and ARGs can spread through water systems, potentially reaching human populations and reducing the effectiveness of standard antibiotic treatments. This exacerbates the global health crisis of antimicrobial resistance, leading to higher medical costs, prolonged hospital stays, and increased mortality. The emergence of ARGs and ARBs is recognized as one of the most critical threats to global health. These genetic elements can spread through microbial communities in aquatic systems, which are amplified by anthropogenic activities such as agriculture and aquaculture, where antibiotics are used extensively. This creates reservoirs of resistance that can be reintroduced into human systems through food and water, posing significant public health risks [11,12].

Since the COVID-19 pandemic, interest in monitoring microorganisms and viral RNA in wastewater has increased significantly. This focus, combined with concerns about persistent contaminants such as pharmaceuticals, highlights the importance of monitoring wastewater generated in hospitals. Depending on the region of the world, the volume of wastewater per patient can vary significantly, ranging from approximately 156 L/patient/day in India to 2258 L/patient/day in Germany [13]. This high volume of wastewater generation makes its monitoring essential to understand the associated risks, such as the spread of antibiotic-resistant microorganisms, the presence of viruses and pathogenic bacteria, and the environmental and health impacts of these contaminants. In addition, pathogens such as cyanobacteria, which produce harmful cyanotoxins, are becoming increasingly relevant. Eutrophication and climate change have intensified harmful algal blooms, resulting in toxins that bioaccumulate in food webs and pose serious risks to aquatic organisms, humans, and animals [14,15].

In this context, the development of new analytical techniques that can quickly and effectively quantify these contaminants in various water resources is essential. While numerous analytical techniques exist, biosensors have seen a significant surge in development in recent years, emerging as a promising alternatives for contamination and pathogen monitoring. Traditionally recognized for their applications in the biomedical field, biosensors are now being increasingly adapted and optimized to detect pollutants in air, soil, and water [16]. They offer the potential for sensitive, rapid, and cost-effective detection of ECs and pathogens in wastewater and natural water bodies. Emerging techniques such as high-resolution mass spectrometry and advanced biosensors have greatly improved contaminant identification. In addition, bioanalytical tools such as aptamer-based biosensors and molecular diagnostics offer increased sensitivity for the real-time monitoring of chemical and biological contaminants. These advances are crucial to addressing the persistent and evolving challenges posed by emerging contaminants [17,18,19].

For this reason, this review aims to provide a comprehensive and accessible overview of biosensors, making their basic principles, significance, and applications understandable to a wide audience. It highlights their growing role in several aspects including contaminants quantification, as ECs and pathogens in water, while also showcasing recent technological advancements and how they have improved existing detection methods. Additionally, the review explores emerging trends, current challenges, and the future perspectives of biosensors, addressing key innovations and potential solutions that will shape their development in the coming years.

### 1.1. The Role of Biosensors

Traditional methods for detecting ECs and pathogens in wastewater, such as chromatography and culture-based techniques, are often time-consuming, labor-intensive, and require sophisticated laboratory equipment. For this reason, in recent years, there has been a growing interest in the application of biosensors for the quantification of these environmental contaminants [20]. Unlike the traditional analytical methods mentioned above, biosensors offer several advantages, including the following:

- Rapid detection: Biosensors deliver real-time or near-instant results, facilitating faster decision making. They serve as a cost-effective and efficient alternatives to traditionally slow and labor-intensive methods for food and water pathogen detection, making them ideal for large-scale monitoring applications [21].
- High sensitivity and specificity: The incorporation of specific biorecognition elements to biosensors ensure high precision and enhanced sensitivity in the detection of target analytes. For instance, aptamer-based biosensors have proved remarkably efficiency in the identification of pharmaceuticals, heavy metals, endocrine-disrupting chemicals, and agricultural contaminants in environmental samples [22].
- On-site monitoring: The portability of biosensors allows on-site monitoring, which reduces the need to transport samples and allows immediate analysis. For example, enzyme-based electrochemical biosensors have been developed for microfluidic applications, facilitating the in situ detection of contaminants [21].

**Figure 2:**
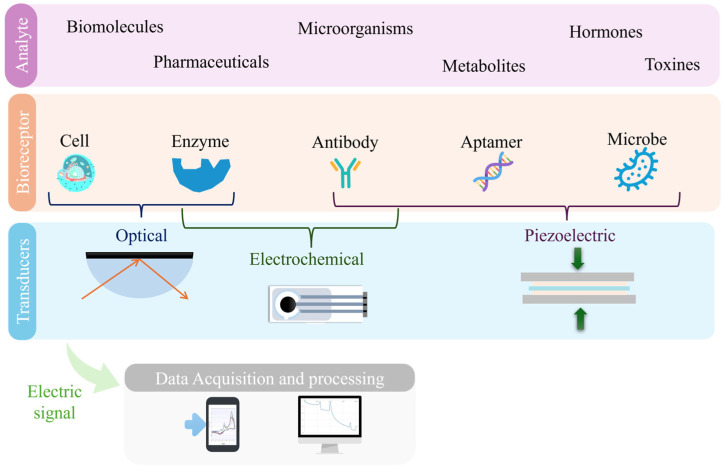
Simplified schematic illustrating the key components of a biosensor.

Based on these benefits, biosensors have become powerful tools for monitoring the presence in wastewater of contaminants and pathogens, contributing substantially to safeguard human health and the environment [23]. Therefore, understanding their nature, structure and function is essential to appreciating their impact. Essentially, they are devices that integrate a biological recognition element with a transducer that converts the resulting physicochemical reaction into a measurable signal [24,25]. These devices usually comprise a bioreceptor, a transducer, and a signal processor as main components (Figure 2) [26].

The bioreceptor, a crucial component, is typically composed of a layer of macromolecules as antibodies, nucleic acid probes, or viral proteins, specifically designed to bind to the target analyte [27]. This interaction generates a signal, which is then captured by the transducer and converted into an electrical response [20,24,25]. The processed signal is subsequently analyzed and displayed as a quantifiable digital output, allowing for the accurate and real-time detection of contaminants.

To enhance the interaction between the analyte and the bioreceptor, additional modifications are often required. One notable example is the integration of nanomaterials, which significantly improves biosensor performance by increasing sensitivity [28,29]. Nanomaterials, due to their high surface area and unique physicochemical properties, enhance the interaction between biosensors and target pathogens, leading to greater sensitivity and selectivity in detection [30]. The incorporation of nanomaterials also enables biosensors to achieve faster response times, which is critical for the timely detection of pathogens in water samples [31].

The necessity of rapid contaminant detection is evidenced in cases where timely identification has enabled effective interventions. Wu et al. developed a whole-cell biosensor for detecting *Pseudomonas aeruginosa* and *Burkholderia pseudomallei*, two key waterborne pathogens [32]. Using a QscR quorum sensing system in an *E. coli* host, this biosensor expresses an enhanced green fluorescent protein upon detecting quorum sensing molecules, facilitating cost-effective and rapid detection. Unlike traditional methods, which require hours or days, this biosensor provides results within minutes, allowing for swift responses to contamination events [33].

Similarly, the COVID-19 pandemic highlighted the urgent need for rapid and accessible diagnostic tools. While reverse transcription polymerase chain reaction (RT-PCR) remains the gold standard, it requires specialized equipment, trained personnel, and long processing times, limiting its practicality for large-scale screening. In contrast, biosensors have emerged as fast, sensitive, and on-site alternatives [34]. Technologies such as aptamer-based biosensors, which utilize single-stranded DNA or RNA molecules to selectively bind to SARS-CoV-2 proteins, have demonstrated high specificity and efficiency, offering a reliable mechanism for real-time virus detection [4,35].

For example, electrochemical biosensors have been developed to detect SARS-CoV-2, the virus responsible for COVID-19. These biosensors provide benefits such as speed, accuracy, portability, and real-time results. They can detect viral components, such as proteins or nucleic acids, directly from patient samples, significantly reducing the time required for diagnosis [36]. A notable innovation is the use of a DNA biosensor leveraging Au@Pt/Au core@shell nanoparticles, as described by Martínez-Periñán et al. [37]. These nanoparticles serve as dual-function components, combining excellent electrocatalytic activity for the oxygen reduction reaction with their ability to bioconjugate with thiolated DNA probes. The resulting biosensor demonstrated a low detection limit of 32 pM for SARS-CoV-2 RNA and facilitated the direct analysis of patient samples without requiring amplification processes. This approach provided a robust and highly sensitive platform for detecting viral RNA, yielding results comparable to RT-qPCR but with a significantly reduced processing time. Such advancements exemplify the growing potential of biosensors to deliver rapid, reliable, and scalable diagnostic solutions in public health crises.

Nanobiosensors incorporating nanomaterials have significantly enhanced the sensitivity and specificity of COVID-19 detection, enabling early diagnosis and timely intervention. The integration of nanotechnology has led to the development of fast, accurate, and cost-effective point-of-care devices, particularly beneficial in resource-limited settings [38]. For instance, carbon-based nanomaterials like graphene improve electrical conductivity and signal amplification, achieving unprecedented sensitivity in detecting RNA or viral antigens [18,39].

The application of biosensors in the diagnosis of COVID-19 exemplifies their potential to revolutionize pathogen detection, providing a means for rapid and reliable diagnosis that is crucial for controlling infectious disease outbreaks and safeguarding public health [40]. These advances not only highlight the versatility of biosensors, but also underscore their role in real-time epidemiological surveillance, enabling proactive measures to be taken during pandemics and reducing the burden on healthcare systems [41,42]

### 1.2. Bioreceptor Immobilization: The Critical Step in Biosensor Development

The immobilization of the bioreceptor onto the transducer is one of the most critical steps in biosensor development, as it directly influences the sensor’s sensitivity, specificity, and overall performance. Effective immobilization is essential to ensure that the bioreceptor retains its biological activity while achieving a stable and robust attachment to the transducer surface. The main challenge lies in maintaining a delicate balance between achieving strong immobilization and preserving the native functionality of the bioreceptor, such as enzyme activity, antigen–antibody recognition, and nucleic acid hybridization. Some of the most commonly employed immobilization techniques include adsorption, covalent bonding, cross-linking, and entrapment [43,44,45,46].

The first technique, adsorption, relies on the physical adsorption of the bioreceptor onto the surface of the transducer via van der Waals forces, hydrogen bonding, or electrostatic interactions. This method is simple and cost-effective; however, it may lead to disruption or the loss of activity under changing environmental conditions. Another widely used technique involves covalent bonding and its related method, cross-linking. Both are based on covalent interactions, but the key difference lies in how the interaction occurs. Covalent bonding directly links the bioreceptor to the transducer through functional groups such as amines, carboxyls, or thiols, while cross-linking uses bifunctional reagents to form a stable network between the bioreceptor and the transducer.

Another important immobilization technique is entrapment and microencapsulation, where the bioreceptor is physically enclosed within a polymeric matrix or membrane. This method ensures that the bioreceptor remains near the transducer, providing a protective environment. However, it may limit substrate accessibility and diffusion, potentially reducing the overall efficiency of the biosensor. In the case of covalent bonding, the bioreceptor is directly attached to the transducer through specific functional groups, such as amines, carboxyls, and thiols. This method ensures strong and stable immobilization, minimizing the risk of bioreceptor leakage. However, precise control is required during the process to prevent potential damage that could compromise the biological activity of the bioreceptor.

On the other hand, cross-linking employs bifunctional reagents that act as intermediaries to facilitate the formation of covalent bonds between the bioreceptor and the transducer. These agents are essential because without them, the bond between these two elements would not occur. While this technique provides excellent stability, improper cross-linking can reduce the biological activity of the bioreceptor, thereby affecting the overall performance of the biosensor.

The selection of an immobilization method relies on the bioreceptor type, transducer material, and biosensor application. Each one presents advantages and drawbacks, requiring optimization to ensure maximum performance and reliability. The enhancement of the immobilization strategies improves the biosensor analytical capabilities for their use in environmental monitoring, medical diagnostics, and industrial applications.

### 1.3. Types of Biosensors

As discussed in Section 1.1, biosensors are sophisticated analytical devices that integrate a biological recognition element with a physicochemical transducer to detect specific analytes (Figure 2).

The biological recognition component, often referred to as a bioreceptor, may include entities such as enzymes, antibodies, aptamers, or even tissues and whole-cells, each selected for its specific interaction with the target analyte. In some cases, bioreceptors can be derived from microbes, which encompass bacteria, viruses, protozoa, fungi, and archaea, due to their ability to bind selectively to specific molecules or structures. After interacting with the analyte, the bioreceptor undergoes a biochemical change, which the transducer converts into a measurable signal, either electrical, optical, or mechanical [47,48]. This conversion facilitates the quantification or qualitative evaluation of the presence of the analyte [49]. The design and performance of biosensors are heavily determined by the characteristics of the bioreceptor and the transduction and immobilization techniques [27]. Consequently, biosensors are commonly classified based on two fundamental components: the type of biorecognition element and the detection technique employed. The detection techniques, which include electrical/electrochemical, optical, and piezoelectric methods, are implemented through various transducers such as electrodes, optical fibers, and oscillators. This classification framework not only aids in understanding the operating principles of different biosensors but also guides their application in diverse fields, including medical diagnostics, environmental monitoring, and food safety [50].

## 2. Biorecognition Elements

The biorecognition element is a fundamental component that directly influences the specificity, sensitivity, and functionality of the biosensor [51]. Selecting the right biorecognition element is crucial to ensure optimal performance in various applications, including healthcare, environmental monitoring, and food safety. The main types of biosensors categorized by their biorecognition elements include enzymes, antibodies, DNA, whole cells, aptamers, antimicrobial peptides, and artificial binding proteins [51,52].

### 2.1. Enzyme-Based Biosensors

They use enzymes to catalyze specific biochemical reactions with the target analyte, generating a measurable signal [53]. Many enzymes can interact with a wide range of substrates. The selectivity of enzyme-based biosensors varies and is influenced by factors such as the sensor design, the detection technique, and key elements such as the immobilization matrix, the operating conditions, and the specific enzyme used [54].

**Figure 3:**
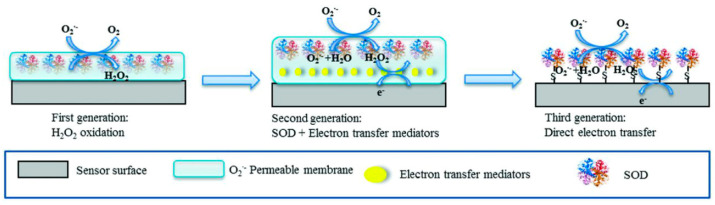
Principle of detection and the generational evolution of superoxide biosensors utilizing the superoxide dismutase enzyme. The figure illustrates the progression from first-generation biosensors, relying on direct enzyme–substrate interactions, to second- and third-generation biosensors, which incorporate advanced materials and electron transfer strategies to enhance sensitivity, specificity, and stability. Reprinted with permission from Liu et al. [42]. Copyright 2015 American Chemical Society.

These biosensors are characterized by several properties such as (i) high specificity for its substrates, which allows the accurate detection of analytes such as glucose and urea; (ii) wide applicability in clinical diagnosis; and (iii) extended utility in environmental monitoring, detecting toxins such as organophosphates and heavy metals [55]. Thus, enzymes such as laccase and tyrosinase are used to detect phenolic compounds in wastewater [56,57] or radical species such as superoxide, as illustrated in Figure 3 below.

Enzyme-based biosensors have always represented a promising technology for environmental monitoring, particularly in the assessment of water quality and the detection of pollutants [58]. The functionality of these biosensors is based on the principle of enzymatic inhibition, which involves enzymes such as urease, horseradish peroxidase, tyrosinase, and cholinesterase [59]. Certain pesticides, such as organophosphates and carbamates, inhibit enzymes like acetylcholine esterase by binding to their active sites, thereby preventing the hydrolysis of acetylcholine into acetate and choline [60]. This enzymatic inhibition has been widely utilized as a reliable mechanism for detecting these toxic substances in environmental samples. Early works in this field include the study by La Rosa et al. [61], who demonstrated an acetylcholinesterase-based amperometric biosensor using 4-aminophenyl acetate as a substrate. This approach achieved the highly sensitive detection of organophosphorus and carbamate pesticides by monitoring the inhibition of enzymatic activity. Similarly, Abad et al. [62] developed a piezoelectric biosensor for pesticide detection, which relied on frequency changes induced by enzymatic reactions on immobilized acetylcholine esterase. The system achieved remarkable detection limits, as low as 5.0 × 10⁻^2^ µM for paroxon.

The integration of these enzyme systems with electrochemical and piezoelectric transducers offers several advantages, including rapid, in situ analysis with minimal sample preparation, representing a significant improvement over traditional laboratory-based methods [63]. By combining the specificity of enzyme inhibition with the sensitivity of modern transducers, these biosensors provide a promising tool for environmental monitoring of pesticide residues.

Recent advancements in biosensor technology have led to the development of more innovative and practical solutions for organophosphate detection. For instance, Mool-am-kha et al. [64] introduced a fluorescence-based biosensor to detect organophosphates, such as malathion, by leveraging the inhibition of alkaline phosphatase. The biosensor converts fluorescence signals into RGB values using a portable device connected to a smartphone, achieving a detection range of 0.30–3.03 µM with a limit of detection as low as 0.15 µM. This system demonstrated high precision in vegetable sample analysis, offering a cost-effective and portable solution for the real-time monitoring of pesticide residues. Similarly, Miglione et al. [65] developed a portable electrochemical biosensor integrated into gloves for the detection of organophosphates, such as dichlorvos, directly on fruit peels [58]. This innovative design employs butyrylcholinesterase as the bioreceptor, whose activity is inhibited by organophosphates, achieving a detection limit in the nanomolar range with excellent repeatability. The glove-integrated sensor offers a practical and efficient tool for on-site monitoring, particularly in precision agriculture.

For the detection of herbicides such as triazines and phenylureas, which disrupt the photosynthetic process, biosensors have been developed that incorporate chloroplast or thylakoid membrane receptors, as well as unicellular algae [66,67]. These biological components enable direct interaction with herbicides, facilitating sensitive detection. Additionally, enzymes such as urease, organophosphorus hydrolase, and cholinesterase continue to play a critical role in the design of electrochemical biosensors for pesticide detection [68].

To detect heavy metals, enzyme-based biosensors have proven effective using the inhibitory effects of metals, such as copper, lead, and cadmium, on enzymatic activity to form the basis for their detection [69]. Additionally, enzymes such as laccase and tyrosinase facilitate the oxidation and detection of phenolic compounds [69]. This approach not only demonstrates the versatility of enzyme-based biosensors for detecting specific analytes but also underscores their usefulness for monitoring toxic substances such as mercury in environmental samples [70]. Yang et al. [71] developed a potentiometric biosensor for mercury detection, utilizing the inhibition of the urease enzyme as a detection mechanism. The biosensor demonstrated a linear detection range of 0.09–1.99 µM and achieved an impressive detection limit of 0.05 µM. This biosensor works based on the enzymatic hydrolysis of urea, which produces ammonia and carbon dioxide, causing an increase in the pH of the solution. In this case, it is necessary to use a pH-sensitive polyvinyl chloride (PVC) membrane electrode to monitor changes in the concentration of hydrogen ions. As the urea concentration increases, the potential response of the biosensor changes, accordingly, allowing the quantification of urea levels [72].

Advances in nanotechnology have further enhanced the capabilities of enzyme-based biosensors. The incorporation of nanoparticles with enzymes not only significantly improves their stability but also increases the active electrode surface area, thereby enhancing their sensitivity [73]. Nanostructured materials, such as metal nanoparticles and metal oxides, have been effectively combined with immobilized enzymes to detect contaminants such as nitrates and pesticides in water samples. These hybrid systems achieve high accuracy and low detection limits, underscoring their potential for fast and reliable environmental monitoring [59].

Despite these advances, significant challenges remain. Enzyme stability in complex matrices, such as those with varying pH or ionic strength, can compromise sensor performance. In addition, the interference of non-target compounds in environmental samples can reduce the detection accuracy [74]. Addressing these limitations requires ongoing research in enzyme immobilization techniques (Section 1.2), the use of extremozymes to improve durability, and the integration of biosensors with advanced technologies.

Extremozymes, derived from thermophilic organisms, offer unique advantages to overcome these challenges due to their exceptional stability under adverse conditions. A series of specific structural characteristics of thermostable enzymes prevents denaturation at high temperatures and maintains enzymatic activity [75]. These adaptations include an increased number of salt bridges and hydrogen bonds between side chains, improved hydrophobic interactions, and a more rigid overall structure. In addition, thermophilic proteins typically exhibit a higher content of Arg and Tyr residues and fewer thermolabile residues, such as Asn and Gln, compared to their mesophilic counterparts.

Beyond thermal stability, extremozymes resist proteolysis and demonstrate high chemical and pH stability, which makes them ideal candidates for applications in biosensors designed for environmental monitoring. Recent developments have shown that thermophilic microorganisms, such as bacteria and fungi, are a significant source of novel catalysts of industrial interest. These extremozymes can replace mesophilic enzymes in applications that require high-temperature processes, reducing the risks of microbial contamination, improving the solubility of the substrate and improving reaction rates. In addition, the heterologous overexpression of extremozymes in *Escherichia coli* (*E. coli*) facilitates the production of large amounts of enzymes, which are easily purified by heat treatment.

By integrating extremozymes into biosensor designs, researchers can achieve robust performance and reliability, even under challenging environmental conditions. This integration represents a critical step forward in the development of biosensors capable of accurately detecting long-term contaminants in complex environmental matrices.

By leveraging enzyme inhibition principles, advances in nanotechnology and innovative sensor designs, enzyme-based biosensors provide a powerful and adaptable solution for long-term environmental monitoring. Its development continues to bridge the gap between laboratory-based methods and field-ready technologies, ensuring cost-effective and reliable contaminant detection.

### 2.2. Antibody-Based Biosensors

Also known as immunosensors, antibody-based biosensors take advantage of the high specificity of antigen–antibody interactions to detect pathogens, toxins, and biomarkers. These biosensors are essential tools in medical diagnosis and food safety. Recent advances, such as the development of monoclonal and recombinant antibodies, have significantly improved the reproducibility and specificity of immunosensory assays. In addition, its multiplexing capabilities allow the simultaneous detection of multiple antigens, which is particularly valuable in complex diagnostic scenarios, including the analysis of cancer biomarkers and the monitoring of multiple pathogens. These characteristics underline the fundamental role of immunosensors in the advancement of precision medicine and the guarantee of public health safety [76].

Most biosensors of this type commonly use self-assembled monolayers (SAMs) to enhance the immobilization of antibodies. Essentially, a SAM is an organized molecular film that spontaneously forms on the surface of a solid material, typically a metal such as gold but can be other material as fluorine-doped tin oxide thin film [77]. Its main advantage lies in providing controlled functional surfaces, which improve the immobilization of biomolecules (such as antibodies or enzymes) and facilitate biological interactions [78,79].

**Figure 4:**
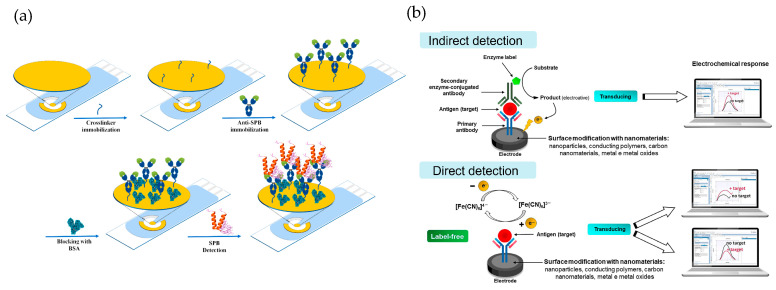
(**a**) Schematic representation of the SAM formation process in antibody-based biosensors. This diagram is adapted from the work of Ben Messaoud et al. [86]. Copyright 2023, Elsevier. (**b**) overview of electrochemical biosensor detection strategies, featuring indirect detection (top) with enzyme-labeled antibodies and direct detection (bottom) via label-free antigen recognition, both enhanced by nanomaterial surface modifications. This diagram is adapted from the work of Ferreira et al. [87], copyright 2023 MDPI (Basel, Switzerland).

As schematically shown in Figure 4a, SAMs typically incorporate chemical compounds with an affinity for the material’s surface, forming covalent or semi-coordinated bonds. These compounds contain functional groups such as thiols, amines, acids, disulfides, and silanes [80,81]. For example, in gold electrodes used as working electrodes, thiol-based chemistry is commonly employed. To facilitate the immobilization process, linking molecules such as glutaraldehyde can be used as auxiliaries in this step. Another strategy involves using polymeric matrices with reactive functional groups (amino and hydroxyl), which are suitable for immobilizing biomolecules [82]. As the final step in the synthesis, bovine serum albumin (BSA) is frequently used to block the electrode surface, preventing non-specific interactions [83,84]. This step contributes to improving the selectivity and accuracy of the system.

As illustrated in Figure 4b, antibody-based biosensors (also known as immunosensors) can incorporate nanomaterials to enhance recognition and amplify the obtained signal, as demonstrated by the work of Chang et al. [85]. In that study, the authors developed an ultrasensitive biosensor for the detection of clinical biomarkers, using prostate-specific antigen (PSA) as an example—a biomarker associated with prostate cancer. The immunosensor employs a sandwich-type reaction based on biotinylated antibodies. A primary antibody is immobilized on a gold electrode modified with SAM, while a secondary antibody functionalized with a metal–organic framework (MOF) loaded with pyrroloquinoline quinone (PQQ) enables signal amplification. PSA detection is achieved through a redox cycling system that uses PQQ as a catalyst to accelerate the oxidation of tris(2-carboxyethyl)phosphine, a chemical reducing agent. This design facilitates the detection of PSA with high sensitivity (LOD: 2.94 × 10^−11^ µM) and specificity, thanks to the biotin-streptavidin interaction and the use of MOFs, which minimize interference and generate a precise electrochemical signal.

On the other hand, a remarkable example of the use of biosensors in environmental applications, specifically for seawater analysis, is the one developed by Ben Messaoud et al. [88]. Their device employs monoclonal antibodies specific to *Aeromonas salmonicida*, immobilized on a gold electrode through covalent bonding. The design incorporates an integrated electrochemical chip that, using the technique of electrochemical impedance spectroscopy (EIS), enables the ultrasensitive detection of this bacterium. The biosensor can detect the presence of *Aeromonas salmonicida* at concentrations as low as one colony-forming unit per milliliter (1 CFU/mL) directly in seawater, demonstrating exceptional sensitivity and specificity for applications in aquaculture and environmental monitoring.

### 2.3. DNA-Based Biosensors

Highly versatile analytical devices that use nucleic acid sequences to detect complementary strands of DNA or RNA, making them indispensable in various genetic and diagnostic applications. Its importance lies in its ability to facilitate mutation detection, pathogen identification, and forensic analysis, addressing critical health and safety needs. Recent advances, such as the integration of CRISPR-Cas systems, have revolutionized these biosensors by allowing the detection of highly precise and specific nucleic acids, even at low concentrations [89]. In addition, DNA biosensors have been proven to be fundamental in decentralized test scenarios, particularly for epidemic monitoring and zoonotic disease detection. Its fast and accurate results significantly reduce response times during outbreaks, offering invaluable support for public health interventions.

However, in the field of water quality monitoring, these biosensors are increasingly being used for the quantification of heavy metals. This is primarily due to their operating principle, which is based on the affinity of certain metal ions for forming stable structures with specific DNA bases [90,91]. For instance, mercury ions (Hg^2^⁺) can form specific bonds with thymine base pairs, while silver ions (Ag⁺) preferentially interact with cytosine, generating highly stable metal-specific structures under aqueous conditions [92,93]. This behavior enables the development of DNA-based biosensors that leverage this affinity to detect and quantify trace amounts of metals in water with high selectivity and sensitivity. Moreover, the use of DNA as a sensing element not only provides chemical specificity but also offers thermal stability and ease of modification to optimize the biosensor’s performance across different environmental matrices [92].

### 2.4. Whole-Cell Biosensors

Unique analytical tools that use living cells to detect and quantify analytes, offering remarkable versatility in environmental monitoring, healthcare, and industrial applications. These biosensors take advantage of the natural biochemical and physiological responses of cells, which allows the real-time detection of pollutants and their bioavailability. For example, designed bacterial biosensors that emit luminescence in the presence of heavy metals such as mercury and arsenic provide critical information about water quality and ecosystem health [94]. Advances in synthetic biology have significantly expanded its functionality, allowing the design of cells with genetic circuits that respond to specific emerging pollutants, such as endocrine disruptors, through fluorescent or colorimetric signals [95]. These biosensors also play a vital role in assessing the cumulative effects of pollutant mixtures, offering information on their synergistic impacts on cellular metabolism and DNA integrity. Although challenges such as cell viability and reproducibility persist, continuous innovations in encapsulation, microfluidic, and machine learning techniques promise to improve their stability and analytical accuracy. As tools for comprehensive pollution monitoring and impact assessment, whole-cell biosensors are invaluable for addressing modern environmental challenges and promoting sustainable solutions [96].

Building on this versatility, novel applications of whole-cell biosensors for detecting specific environmental contaminants can be explored. For instance, a transcription factor-based biosensor has been developed by Dey et al. [97] for the detection of Sodium Dodecyl Sulfate (SDS), a widely used anionic detergent known for its harmful effects on aquatic ecosystems. This biosensor employs *Pseudomonas aeruginosa* PAO1 as the chassis organism, utilizing the sdsB1 activator protein and the SDS-responsive sdsA1 promoter to regulate the expression of green fluorescent protein as a transducer. The system demonstrates a linear response in the range of 1.39 to 216.7 µM SDS, with a detection limit of 0.35 µM, showcasing high specificity and minimal interference from other surfactants or metal ions.

### 2.5. Aptamer-Based Biosensors

**Figure 5:**
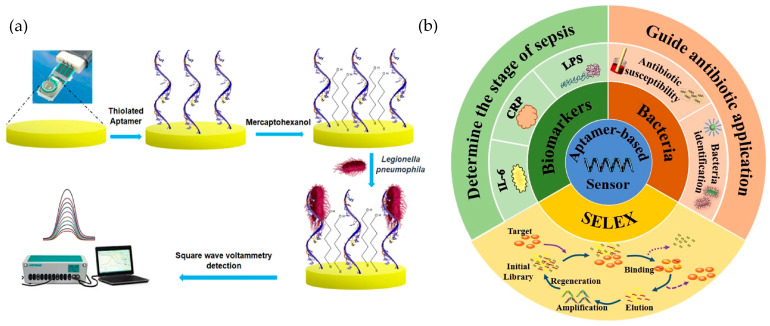
(**a**) Illustration of a biosensor construction process for *Legionella pneumophila* detection, using thiolated aptamers immobilized on a gold electrode. Adapted with permission from Shaukat et al. [101], copyright 2024; Springer Nature. (**b**) Schematic representation of the SELEX process and its applications in aptamer-based biosensors for sepsis detection, targeting biomarkers (CRP, IL-6, LPS) and bacteria to guide antibiotic use. Reproduced with permission from Lui et al. [103], copyright 2021; Springer Nature.

Aptamers are short single-stranded nucleic acid sequences (DNA or RNA) that adopt unique three-dimensional structures, enabling them to selectively bind to a wide variety of targets, such as proteins, small molecules, and even entire cells [98]. Compared to traditional antibody-based biosensors, aptamer-based systems offer significant advantages, including high affinity and specificity, enhanced chemical and thermal stability, and lower production costs. Aptamers can also be chemically modified to improve their resistance to enzymatic degradation, enhancing their performance in complex environmental matrices [99,100]. A clear example of the synthesis methodology for an aptamer-based biosensor is shown in Figure 5a, where a biosensor construction process for *Legionella pneumophila* detection, using thiolated aptamers immobilized on a gold electrode is represented [101]. It consisted of the immobilization of a thiolated aptamer on a gold screen-printed electrode by self-assembly, followed by blocking the gold-free sites with mercaptohexanol. Detection was based on the specific binding of *L. pneumophila* to the immobilized aptamer, which generated an impedance in the charge transfer of the redox probe, monitored by square wave voltammetry.

Recent advancements in systematic evolution of ligands by exponential enrichment (SELEX, Figure 5b) technology have further refined aptamer selection processes [99,102]. For example, Zhang et al. [102] introduced a microfluidic-based SELEX system, which enables faster and more efficient aptamer screening for small molecules, such as advanced glycation end products, significantly reducing the time and cost associated with traditional SELEX methods [92]. These platforms streamline the SELEX process while allowing the real-time monitoring of enrichment rates, ensuring the development of high-quality aptamer candidates for biosensor applications.

A study by Zandieh et al. [104] highlights the selection of specific DNA aptamers for detecting microplastics, particularly PVC and polystyrene (PS). Using the SELEX method, the authors isolated DNA sequences enriched in pyrimidines, predominantly cytosine (50%) and thymine (37%), with minimal purine content. The most effective sequence, named PVC1, exhibited an exceptional binding affinity to PVC, demonstrating a six-times-higher efficiency compared to a random DNA sequence of the same length. The recognition mechanism relies primarily on van der Waals forces, enhanced by the aptamer’s flexible structure, which optimizes surface contact with the plastic. The aptamer PVC1 achieved selective detection of PVC and PS with detection limits of 8.0 µmol and 5.76 µmol, respectively. Using a fluorescence-based assay, the researchers successfully distinguished microplastics even in complex samples, such as wastewater, without interference from other materials like silica. This work offers a sensitive and robust method for environmental monitoring, providing a reliable solution for detecting and quantifying microplastics in real-world scenarios.

In the field of pesticide detection, Feng et al. [105] developed a fluorescence-based aptasensor for detecting acetamiprid, a neonicotinoid pesticide. The biosensor uses oxidized carbon nanohorns as the signal amplification platform and an aptamer as the biorecognition element. The system achieved an impressive detection limit as low as 2.84 × 10^−8^ µM and demonstrated high selectivity, with no significant interference from other pesticides. The signal amplification was enhanced with cryonase enzymes, further improving the sensitivity of the detection system. Similarly, Thai Duong et al. [106] introduced a label-free biosensor based on liquid crystals for detecting acetamiprid. In their system, the binding of the pesticide to its specific aptamer induces changes in the orientation of the liquid crystals, producing measurable optical signals. The sensor achieved an exceptional detection range of 5 × 10^−6^ µM to 0.070 µM, with a detection limit as low as 0.69 × 10^−6^ µM.

### 2.6. Antimicrobial Peptide (AMP)-Based Biosensors

These sensors utilize natural immune defense molecules to detect bacteria and other pathogens with remarkable efficiency. Their fast-binding kinetics and broad specificity make them ideal for applications that require fast and accurate microbial detection. AMP are commonly integrated into impedimetric biosensors, enabling sensitive, real-time bacterial detection for clinical diagnosis and food safety monitoring. In addition, its relatively simple structure allows for extensive customization, facilitating the design of biosensors adapted to specific applications and target analytes. These attributes position AMP-based biosensors as a valuable tool to address critical challenges in healthcare, food safety, and environmental monitoring [106].

### 2.7. Biosensors Based on Artificial Binding Proteins

Biosensors utilizing artificial bindings proteins, such as domain antibody-related proteins and non-immunoglobulin scaffold proteins, overcome several limitations of traditional antibodies, offering greater stability and scalability for large-scale applications [107]. These modified proteins are designed for the rapid and precise detection of a wide range of analytes, from small molecules to complex proteins, ensuring high specificity and sensitivity. Their robustness and adaptability have allowed their integration into portable devices, which makes them very suitable for point-of-care diagnostics and field applications. These features underscore the transformative potential of biosensors based on artificial binding proteins to advance healthcare accessibility, environmental monitoring, and real-time analytical solutions [108].

These diverse biorecognition elements highlight the adaptability and accuracy of biosensors in a variety of applications. Leveraging advances in biotechnology and synthetic biology, biosensors are becoming indispensable tools to address critical challenges in the fields of healthcare, environmental monitoring, and food safety. Continued research and innovation promise to expand their capabilities and strengthen their role in solving modern analytical challenges.

## 3. Transduction Mechanism

Both the bioreceptor and the transducer employed are fundamental components for determining the sensitivity, specificity, and suitability of the biosensor for various applications. The transducer, in particular, is responsible for converting the interaction into a measurable signal. The primary transduction mechanisms include electrochemical, optical, and piezoelectric methods.

Regarding the first type of transducer, it is based on biochemical interactions being transformed into measurable electrical signals. This process relies on detecting changes in electrical properties, such as current, potential, and conductance, resulting from specific chemical reactions between the analyte and the bioreceptor. For this reason, these transducers are typically classified based on the type of electrical signal they measure, and the technique used for detecting biochemical interactions, with the most used types being amperometric, potentiometric, conductometric, and impedimetric transducers.

For optical transducers, this interaction is converted into measurable optical signals based on changes in the properties of light, such as intensity, phase, polarization, and wavelength. Therefore, the most common classification of this type of transducer is based on the optical principle they employ. The most widely used optical transducers in biosensors include those based on surface plasmon resonance (SPR), fluorescence, and absorbance as measurement techniques.

As for the piezoelectric transducer, it is primarily based on the mass change occurring on the surface of the piezoelectric crystal, which oscillates at a specific frequency depending on this mass. Thus, the frequency change is proportional to the amount of analyte present. Among these techniques, the most well-known and widely used transducer is the Quartz Crystal Microbalance (QCM), but surface acoustic wave and bulk acoustic wave devices can also be employed in biosensors.

### 3.1. Electrochemical Biosensors

**Figure 6:**
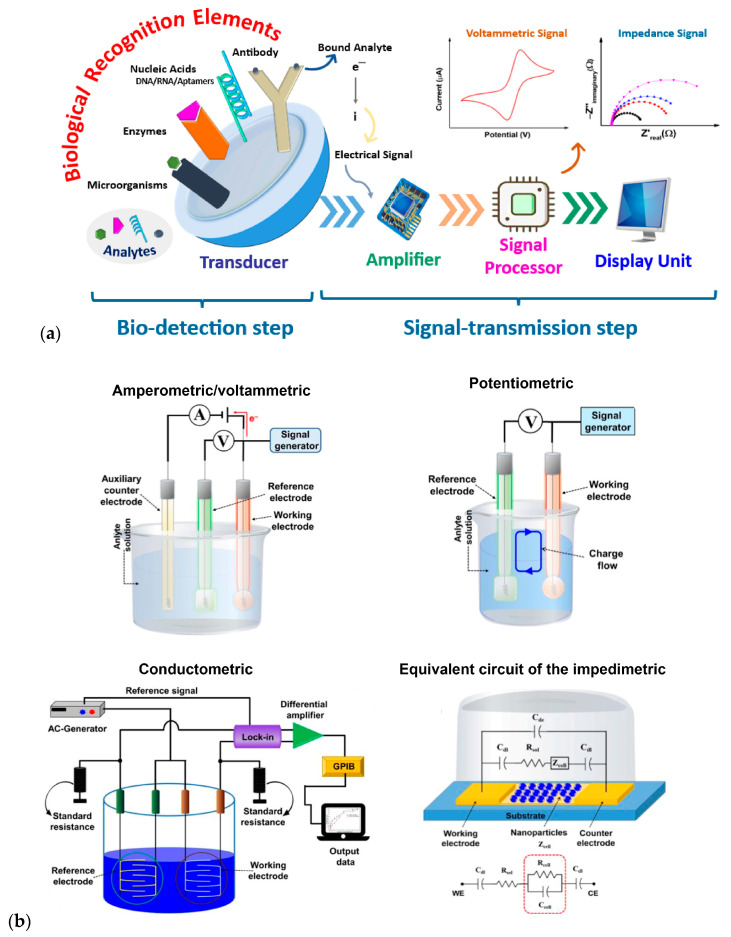
(**a**) Stages in the functional mechanism of an electrochemical biosensor. This diagram is reprinted from [53], copyright 2023, with permission from Elsevier. (**b**) Schematic diagrams of various electrochemical transducers. This diagram was adapted from Naresh and Lee [47]. Copyright 2021 MDPI (Basel, Switzerland).

Electrochemical biosensors, known for their ability to detect biochemical reactions produced by one or more analytes through discrete or continuous signals, are highly sensitive and versatile, making them ideal for numerous applications (Figure 6a). These devices operate based on different electrochemical principles, such as amperometry, potentiometry, conductometry, and impedance spectroscopy (Figure 6b) [53].

As early as 1956, Professor Leland C. Clark, Jr., known as the biosensor concept creator, introduced the use of electrochemical biosensors with the publication of his article on oxygen electrodes for the continuous measurement of arterial oxygen tension [109]. This discovery laid the groundwork for the proposal that Clark and Lyons published in 1962, attempting to expand the range of analytes that could be measured through so-called “enzyme electrodes” [110,111]. In this study, glucose oxidase was immobilized on an oxygen electrode using a dialysis membrane, with a proportional relation between the decrease in oxygen and the glucose concentration. The significance of Clark’s design was so notable that many studies continue to use the oxygen measurement concept [112].

Today, advances in micro- and nanoscale manufacturing technologies have facilitated the development of electrochemical biosensors designed for point-of-care use [113]. This progress has allowed the miniaturization and integration of complex systems into portable and easy-to-use tools [114]. For example, conventional potentiostats, traditionally large and limited to the laboratory, have been transformed into compact devices the size of a cell phone. Despite their small size, these miniaturized potentiostats retain all their functionality as electroanalytical instruments, allowing their deployment in the field with minimal training [115].

#### 3.1.1. Amperometric Sensors

These sensors measure the current resulting from the oxidation or reduction of electroactive species. The produced current is directly proportional to the concentration of the target analyte. Amperometric biosensors are widely used in clinical diagnostics, such as glucose monitoring in diabetes management [116]. Beyond their clinical applications, these biosensors have been explored for the detection of emerging contaminants of diverse nature (Table 1).

A remarkable case is the detection of chlorpyrifos, an organophosphate pesticide widely used in agriculture whose accumulation can cause serious risks to human health and environment [117]. In this framework, amperometric enzyme biosensors are presented as a potential tool for chlorpyrifos detection due to their rapidity, in situ analysis and high sensitivity. These devices detect the current generated by the oxidation of thiocholine at the working electrode, which occurs through the hydrolysis of acetylcholine chloride catalyzed by the enzyme acetylcholinesterase (AChE), immobilized on the electrode. When chlorpyrifos is present, it inhibits the activity of the enzyme, reducing the production of thiocholine and, thus, the current generated, which allows the pesticide to be detected [118].

**Table 1:** Classification of electrochemical biosensors for pathogens and emerging pollutants in terms of target, electrochemical method, working electrode, biorecognition element, and limit of detection (LOD).

Target	Working Electrode	Measurement Technique	Biorecognition Element	LOD	Ref.
**Pathogens**					
*Salmonella*	CoFe_2_SO_4_/SWCNT modified with TEOS/APTES	CV/EIS	Functionalized DNA probe	2.34 × 10^−10^ µM	[119]
*E. coli*	Ag-Carbon	CV	*E. coli*-aptamer	34 CFU/mL	[120]
SARS-CoV-2	Modified PCB	CV/DPV	DNA-aptamer	1.7 × 10^−9^ µM	[121]
**Pharmaceuticals**					
Sulfamethoxazole	Au/SPE	DPV	Tyrosinase enzyme	22.6 µM	[122]
Bevacizumab	OG	DPV	Anti-bevacizumab	0.02 µg/mL	[123]
Lyncomycin Neomycin	Au/CNF/SPE	SWV	DNA-aptamer	4.92 × 10^−8^ µM 5.55 × 10^−8^ µM	[124]
**Pesticides and agrochemicals**					
Atrazine	Au _5_ Ir@RFBP-GQD	CV/DPV	Hairpin DNA	3.4 × 10^−13^ µM	[125]
Chlorpyrifos	SPCE modified with AChE/CS-GO/GO/CNF	SWV	AChE enzyme	2.2 × 10^−3^ µM	[126]
Chlorpyrifos	SPCE modified with CuNWs/rGO	CV	AChE enzyme	8.84 × 10^−3^ µM	[127]
Methyl parathion Chlornitrofen	GC modified with CRL@MAC-ZIF-8/CS	CV/EIS	CRL enzyme	0.06 µM 0.03 µM	[128]
**Endocrine disruptors**					
4 n-nonylphenol 4-t-octylphenol	SPE modified with [BMIM][PF_6_]	CV/EIS	Horseradish peroxidase enzyme	1.1 µM 0.4 µM	[129]
17β-estradiol	Au/NiHCF NPs	DPV	Aptamer	0.8 × 10^−6^ µM	[130]
**Persistent organic compounds**					
BDE-100 PBB-1 PCB-1 PCB-28 PCB-101	Pt/PANI	Amperometry	Horseradish peroxidase enzyme	2.48 × 10^−5^ µM 7.72 × 10^−5^ µM 1.17 × 10^−4^ µM 6.21 × 10^−5^ µM 5.82 × 10^−5^ µM	[131]

The following acronyms are used in Table 1: SWCNTs: single-walled carbon nanotubes; TEOS/APTES: tetraethyl orthosilicate/(3-amonopropyl)triethoxysilane; PCB: printed circuit board; SPE: screen-printed electrodes; GO: graphene oxide; CNF: carbon nanofiber; GQD: graphene quantum dot; AChE: acetylcholinesterase; CS: chitosan; NWs: nanowires; rGO: reduced graphene oxide; GC: glassy carbon; CRL: *Candida rugosa* lipase; MAC-ZIF-8: macro-microporous ZIF-8 nanofibers; [BMIM][PF_6_]: 1-butyl-3metthylimidazolium hexafluorophosphate; PANI: polyaniline; NiHCF NPs: nickel hexacyanoferrate nanoparticles; PBB-1: 2-Bromobiphenyl; PCB-1: 2-Chlorobiphenyl; PCB-28: 2,4,4′-Trichlorobiphenyl; PCB-101: 2,2′,4,5,5′-Pentachlorobiphenyl; BDE-100: 2,2′,4,4′,6-Pentabromodiphenyl ether; CV: cyclic voltammetry; DPV: differential pulse voltammetry; EIS: electrochemical impedance spectroscopy; SWV: square wave voltammetry.

However, the low conductivity of AChE needs to be compensated for by obtaining a functional biosensor, so the use of conductive nanomaterials has become increasingly popular. Thus, the incorporation of nanomaterials in electrochemical biosensors has opened a plethora of possibilities to improve their performance. These nanoscale structures possess unique properties, such as a high surface-to-volume ratio, which provides a larger active area for biomolecular interactions. In addition, nanomaterials can be functionalized with various molecules, which allows the creation of highly selective and sensitive biosensors [132].

For example, Suwannachat et al. [127] performed a simple modification on screen-printed carbon electrodes (SPCEs) with copper nanowires and reduced graphene oxide, improving the electronic properties of an SPCE and facilitating the immobilization of AChE. Similarly, Tun et al. [126] also modified an SPCE with graphene oxide, a graphene oxide–chitosan composite, AChE, and cellulose nanofibers. In both cases, the pesticide detection limit remained in the order of 10^−3^ µM, but Zhimin et al. [118] were able to achieve an LOD in the order of 10^−7^ µM using a biosensor based on the concept of nanoarchitectonics, which allowed them to eliminate the structural and chemical limitations of nanomaterials. Thus, a glassy carbon electrode was modified with nitrogen-doped nanoporous carbon nanocomposites ZnO@CoO hollow core–shell, achieving high values of surface area, graphitization level, and conductivity.

Other examples are the use of enzymes such as laccase and tyrosinase, which are used to detect phenolic compounds in wastewater through amperometric biosensors [131]. These biosensors measure the current changes associated with oxidation or reduction reactions, offering a sensitive method for the detection of contaminants [56].

Advances in electrochemical biosensors have leveraged innovative nanomaterials to improve the detection not only of emerging pollutants, but also pathogens as *E. coli* O157:H7. For example, sandwich-type electrochemical immunosensors incorporating nanocomposites into modified electrodes have shown promising results. In addition, magnetic and bimetallic nanoparticles have been used in point-of-care testing to achieve sensitive and rapid detection [133].

#### 3.1.2. Potentiometric Sensors

Potentiometric sensors measure the potential difference between a working electrode and a reference electrode, with no significant current flow. The measured potential correlates with the logarithm of the analyte concentration, following the Nernst equation. Potentiometric biosensors are commonly used for detecting ions and small molecules [134] and are recognized for their affordability, compactness, and high sensitivity and selectivity. These sensors take advantage of ion-selective electrodes (ISEs) and ion-sensitive field-effect transistors (ISFETs) to effectively collect analytical data [135]. Recent advances have expanded their usefulness in various configurations, including light-addressable potentiometric systems that provide improved spatial and temporal control [136]. For example, a recent innovation presented by Shaibani et al. [137] demonstrated the use of a portable nanofiber light-addressable potentiometric sensor (NF-LAPS) to detect *E. coli* in orange juice. This sensor employed electrospun pH-sensitive polyvinyl alcohol/polyacrylic acid (PVA/PAA) hydrogel nanofibers as the detection layer. Surprisingly, they achieved a detection limit of 100 CFU/mL in one hour, demonstrating a fast and selective performance level. This highlights the potential of light-assisted potentiometric biosensors to meet real-world analytical challenges.

In addition, advances in potentiometric biosensors continue to diversify their applications. Beyond ion detection, they have been integrated with photoelectrochemical systems to improve signal transduction, and molecularly printed polymers to improve analyte specificity and portable configurations for continuous monitoring [136]. Recent advances have explored the use of potentiometric aptasensors integrated with electrogenerated chemiluminescence systems for the detection of *E. coli*. By using SWCNTs with aptamer molecules, these sensors achieve the highly selective detection of bacteria with exceptional sensitivity. The incorporation of Ru(bpy)_3_^2^⁺ modified electrodes enhance signal amplification, enabling the detection of *E. coli* O157:H7 at concentrations as low as 2 CFU/mL [138]. These systems also use innovative fluid flow designs to improve mass transport, facilitating more efficient detection processes even in complex matrices such as seawater [125]. Novel configurations have been developed, such as the incorporation of *Chlamydomonas reinhardtii* algae cells immobilized on carbon black nanomodified SPCE into an electrochemical transduction system [139]. This configuration records the updrafts under light illumination and the applied potential, correlating with the production of oxygen derived from algae in the presence of bacteria *E. coli* [126].

#### 3.1.3. Impedimetric Sensors

Impedimetric sensors are among the first approaches developed for the rapid detection of pathogens. Unlike other electrochemical techniques, they focus on measuring impedance, which reflects changes in the conductivity of the solution or the transfer of electrons on the electrode surface [140]. The most widely used technique for these biosensors is electrochemical impedance spectroscopy (EIS), which applies to analyzing the properties of the system [141]. A notable example is the development of an impedimetric biosensor based on indium tin oxide (ITO) electrodes for the detection of bacteria, including *E. coli*. The immobilization of anti-*E. coli* antibodies on electrodes exhibited high specificity, as evidenced by their ability to distinguish *E. coli* from *Salmonella typhimurium* and other non-specific bacteria [142]. Another breakthrough is the impedance biosensor that incorporates immunomagnetic nanoparticles (MNPs) and urease for the amplification of biological signals [143]. The system combines MNPs with biotinylated antibodies and urease-modified gold nanoparticles, creating a bacteria-specific complex. The impedance decreases as the urease catalyzes the hydrolysis of urea, improving the detection signal.

Recent developments, reported by Cimafonte et al. [144], include an innovative impedimetric biosensor using a simple and cost-effective approach with screen-printed gold electrodes for the rapid detection of *E. coli* in water. The sensor employs a photochemical immobilization technique to covalently attach anti-*E. coli* antibodies to the gold surface, ensuring the vertical orientation of the antibodies for effective interaction. Impedance measurements were performed in 0.01 M phosphate-buffered solution containing Fe(CN)_6_^3−^/Fe(CN)_6_^4−^ 10 mM as a redox probe. Nyquist plots were analyzed using a modified Randles circuit, identifying resistance to charge transfer as a critical parameter influenced by antibody immobilization, bovine serum albumin blockade and *E. coli* binding [144].

In addition to pathogen detection, impedimetric biosensors have been adapted to detect chemical contaminants such as diazinon, an organophosphate pesticide known for its toxicity and persistence in the environment. A novel impedimetric biosensor has been developed using multi-walled carbon nanotubes (MWCNTs) functionalized with poly-L-lysine to immobilize double-stranded DNA (ds-DNA) on the surface of a glassy carbon electrode. This configuration takes advantage of the interaction between diazinon and ds-DNA, resulting in measurable changes in interfacial resistance to charge transfer (Rct). When diazinon intercalates with the DNA helix, it reduces the Rct, creating a reliable signal for quantification. The biosensor demonstrated exceptional sensitivity, reaching a detection limit as low as 3 × 10^−4^ µM and a wide linear dynamic range (0.001–100 μM). Its performance was validated on real samples, including river water and agricultural wastewater, showing high accuracy and recovery rates, underlining its practicality for environmental monitoring [145].

#### 3.1.4. Conductometric Sensors

These sensors assess changes in the electrical conductivity of a solution due to biochemical reactions. The interaction between the biorecognition element and the analyte alters the ionic composition, leading to measurable conductivity changes. Conductometric biosensors are utilized in environmental monitoring and industrial process control [146]. Enzymatic processes are particularly suitable for conductometric biosensors, as they can significantly alter the conductivity and ionic strength of a solution. By incorporating zeolite imidazolate frameworks as an electrolytic material, conductometric devices can further improve their sensitivity and selectivity [147,148]. This approach facilitates the study of kinetics and enzymatic processes, which makes them valuable tools in both research and industrial settings. Conductometric biosensors have found widespread applications in environmental monitoring and industrial process control, where they can be used to detect contaminants, monitor fermentation processes, and evaluate the quality of various products [147].

A notable advance is the use of conductometric immunosensors for the detection of *E. coli* O157:H7 in water. These sensors employ chemically functionalized interdigitated electrodes (IDEs) to improve the binding specificity toward *E. coli* O157:H7. By modifying the electrode surface with 3-aminopropyl triethoxysilane and binding a carboxylated DNA probe specific to the target bacterium, these sensors achieve high sensitivity and selectivity. The interaction between bacterial DNA and the immobilized probe causes detectable changes in electrical conductivity. This method demonstrated an impressive detection range of 1 × 10^−9^ µM to 1 × 10^−3^ µM and a minimum detection limit of only 1 × 10^−9^ µM, making it suitable for early-stage contamination monitoring in water systems [149].

In addition, the integration of advanced microfabrication techniques into IDE biosensors has improved their performance. For example, aluminum-based IDE designs offer advantages such as low cost, high conductivity, and biocompatibility. These features, combined with an accurate photolithographic pattern, allow sensor designs to be optimized to minimize interference and maximize detection capability. These innovations have proven effective in differentiating complementary, non-complementary, and mismatched DNA sequences, ensuring reliable detection even in complex water simples [150,151].

### 3.2. Optical Biosensors

Optical biosensors detect changes in light properties resulting from the interaction between the analyte and the biorecognition element. They offer high specificity and are widely used in various fields. Key techniques include surface plasmon resonance (SPR), ratiometric fluorescence, and Raman spectroscopy.

Furthermore, Surface-Enhanced Raman Scattering (SERS), an enhancement over the Raman technique, is emerging as a potential tool for detecting contaminants and infectious agents in wastewater, as it facilitates the identification of unique vibration patterns associated with biomarkers and chemicals, even simultaneously. This technique is characterized by rapid result acquisition, ease of transport, high sensitivity, and good capability for analyzing complex matrices [152]. However, despite its high sensitivity, this technique has encountered its main limitation in sample preparation and the reproducibility of the generated signal. As a result, numerous strategies have been developed to overcome these challenges, with methodologies based on the creation of well-ordered periodic arrays of metallic nanoparticles standing out [153]. These nanoparticles, especially those made of Ag and Au, significantly amplify the Raman cross-section of molecules close to them due to the strong electromagnetic field induced by localized SPR [154]. This leads to a significant improvement in signal reproducibility and intensity, enabling the detection of individual molecules and trace-level analytes. This was demonstrated in the study developed by Zhao et al. [154], where a core–satellite protein nanostructure was created by assembling BSA onto the core surface of Ag nanoparticle. This arrangement allowed BSA to anchor many triclosan molecules near the surface of the Ag nanoparticles, resulting in amplified SERS signals. The method enabled the detection of triclosan in pond water within 30 min, with an LOD of 0.05 µM.

Table 2 lists some significant examples of detection of pathogens and emerging contaminants in aqueous environments by optical biosensors according to the type of signal used.

SPR is a highly versatile optical technique that measures the changes in refractive index near a metal surface, typically gold or silver, upon binding of an analyte. This tagless method allows the real-time monitoring of biomolecular interactions, providing detailed information on binding kinetics, affinity, and concentration. SPR has become an indispensable tool in drug discovery and molecular biology research due to its ability to provide quantitative and qualitative data without the need for additional labeling [165]. In addition, it is widely used in the study of protein–protein interactions, particularly to determine dissociation constants at antigen–antibody binding. This is crucial to characterize the affinity and specificity during the development of monoclonal antibodies [166]. Advances in SPR technology have expanded its applications to the detection of multiple analytes, significantly increasing the efficiency in biomolecular studies. This expansion has also been applied to the environmental field, enabling the detection of pollutants of different nature, including nanoplastics, which represent one of the most dangerous sources of pollution in water. A recent study of Seggio et al. [164] developed an SPR-based biosensor functionalized with an estrogen receptor as the biorecognition element. To enhance its detection capability, the receptor was coupled to a polymer-based gold nanograting plasmonic platform, achieving a limit of detection LOD of 1.02 × 10^−3^ µg/mL for polymethyl methacrylate, a model nanoplastic. The study utilized simulated seawater as the medium to mimic environmental conditions, reflecting the growing concern over nanoplastic pollution, especially due to the increased production of plastics during the COVID-19 pandemic.

Among the most widely used transducers in biosensors are those based on fluorescence phenomena, which can detect a wide range of analytes through changes in fluorescence intensity. This occurs due to physicochemical changes on the biosensor surface, affecting the light properties that interact with it, when the analyte binds to the biorecognition element. Some examples include variations in the signal produced by the chemical interaction between an antigen and an antibody, and changes in the signal generated by a fluorophore when an interaction occurs between a chain of genetic material (DNA or RNA) and the analyte of interest [167]. However, the main limitation of fluorescence-based platforms lies in the evaluation of a single emission wavelength, which can lead to autofluorescence interferences that compromise the accuracy and reliability of measurements, especially in complex matrices such as food or biological samples. The ratiometric fluorescence method has emerged as an improved solution to address this problem. This technique allows the measurement of variations in emission intensity at two or more different wavelengths, leading to a self-calibration process that effectively reduces interference and improves both the accuracy and biosensor dynamic range [168].

A notable example of this improvement is the competitive ratiometric fluorescence biosensor developed by Huang et al. [158] for the rapid detection of azithromycin. In this setup, thioflavin T (ThT) is used as fluorophore, GO is employed, and an affinity aptamer, selected through magnetic bead-SELEX, serves as the biorecognition element. Briefly, when GO binds to the affinity aptamer, the fluorescence of ThT decreases, while in the aptamer binds to ThT, the fluorescence signal increases. Therefore, if azithromycin is present in the medium, it will bind to the aptamer, preventing its binding to the GO and causing a variation in the output signal, which will be stronger, and achieve an LOD of 9.78 × 10^−3^ µM.

### 3.3. Piezoelectric Biosensors

Piezoelectric biosensors take advantage of the piezoelectric effect, in which certain materials generate an electrical signal in response to mechanical stress, to detect mass changes on the surface of a sensor due to analyte binding. A prominent example is the QCM, which measures frequency changes corresponding to mass variations. The QCM works by oscillating a quartz crystal at a specific frequency; when an analyte binds to the sensor surface, the added mass causes a measurable decrease in the oscillation frequency, which correlates directly with the amount of bound material.

These biosensors are particularly effective for detecting biomolecular interactions, offering real-time and label-free analysis. This capability is invaluable in healthcare applications, including pathogen detection and biomarker analysis [169,170]. For example, piezoelectric immunosensors have been developed for the detection of various pathogens by immobilizing specific antibodies on the sensor surface. After the binding of the target pathogen, the resulting mass change is detected by the piezoelectric sensor, which facilitates rapid and sensitive identification. In addition, piezoelectric biosensors have been used to monitor biomolecular interactions, such as protein–protein and protein–DNA interactions, which are crucial for understanding the mechanisms of the disease and developing therapeutic strategies [171].

The advantages of piezoelectric biosensors include their high sensitivity, fast response times, and the ability to perform real-time and label-free detection, making them suitable for a wide range of applications. However, challenges such as the need for the precise control of sensor surface properties and possible interference from non-specific junctions must be addressed to ensure accurate and reliable measures.

Although no widespread application of piezoelectric biosensors for pollutants detection in water is known, a meaningful example is the regenerative biosensor proposed by Mazouzi et al. [157] for the detection of DFC in river water. Their biosensor used a technique that combined QCM-D and LSPR. In the study, the surface of the quartz crystal was modified by an optimized activation process to allow the efficient binding of DCF, and an anti-DCF monoclonal antibody was immobilized on it. The binding of DCF to the immobilized antibody causes a shift in the standard resonance frequency associated with QCM-D, which allowed the detection of the contaminant with an LOD of 9.49 × 10^−3^ µM. Furthermore, the LSPR technique provides additional information on the coverage and hydration level of the antibody monolayer, which serves as an efficiency indicator and biosensor stability.

## 4. Environmental Real Water Analysis with Biosensors

Environmental sample analyses beyond simple proof-of-principle in ideal conditions have also been performed using biosensors. Numerous publications have focused on the detection of compounds belonging to pharmaceuticals of pesticides in wastewater, river water, and tap water.

Among the most common pharmaceutical groups present in wastewater, and therefore the ones which aroused the greatest detection interest, are the antibiotics employed in treating bacterial diseases [172] such as tetracycline derivatives [173], ampicillin [174,175], kanamycin [176], quinolones [177], and ciprofloxacin [178]. For this purpose, aptamers have been highlighted as the main biorecognition element due to numerous advantages for these types of molecules, such as high specificity, easy synthesis, low cost, and good stability, in contrast with other bioreceptors, such as antibodies and enzymes, which offer high specificity but with significant limitations in terms of stability and reusability [179]. A similar trend is also observed in pesticide detection with aptamer application, especially in the case of organophosphorus compounds, which constitute the largest group of insecticides employed. Thus, aptamers have been applied as a biorecognition element in the case of atrazine [180], imidacloprid [181], acetamiprid and paraquat [182]. However, the main limitation associated with these bioreceptors is the limited interaction between the functional groups of organophosphorus compounds and the aptamer sequences, due to the small molecular structure of the target compounds [183].

All the above-mentioned transduction technologies have found their application in the detection of organic pollutants in water. Optical biosensors show high selectivity, simplicity in operation conditions, stability, and fast response in measurement. However, the detections limits of this technique are conditioned by external factors such as pressure, temperature, and chemical composition of the sample [184]. Similar limitations are found in the case of piezoelectric biosensors, which despite having a high response speed and selectivity, are vulnerable in wet environments or at high temperatures [185], making in situ measurements harder to perform. Regarding electrochemical biosensors, their exceptional sensitivity and high selectivity, as well as their fast response time, are complemented by easy preparation and good portability [186], which has made them one of the most studied alternatives and has attracted the greatest interest in detecting pollutants in real environments, although interference with organic matter and other ions still presents challenges for their application.

Nevertheless, the inclusion of nanomaterials in the design of biosensors has led to a collective and significant improvement in pollutant detection for real water matrices, regardless of their nature. In this way, Talari et al. [187] achieved an LOD lower than 0.01 µM in tap, river, and runoff waters for the detection of diazinon pesticide thanks to an optical apta-nanobiosensor based on reduced graphene quantum dot fluorescence emission, prepared in two steps and with MWCNTs added to improve the detection accuracy. In a similar approach, Hatamluyi et al. [188] succeeded in further increasing the diazinon LOD reaching a limit of 1.02 × 10^−8^ µM in tap and river water by another amperometric apta-nanobiosensor. In this case, it was developed from a zeolitic imidazolate framework-derived nanoporous carbon platform functionalized with aptamers and poured on an SPCE decorated with hierarchical flower-like gold nanostructures. In the field of pharmaceutical detection, a particularly noteworthy case, performed through an electrochemical transduction mechanism, was reported by Roushani et al. [189] in the detection of ibuprofen, reaching an LOD of 9.80 × 10^−8^ µM in wastewater. This ultrasensitive apta-nanobiosensor was designed from nitrogen-doped GQD (N-GQD) and gold nanoparticles (AuNPs) retained over GCE, forming an AuNPs@N-GQD/GCE electrode. Subsequently, the amino capture probe (NH_2_-ssDNA1) was added forming a covalent bond with the AuNPs and the specific ibuprofen aptamer was included. The results show the effective use of cheap and simple apta-nanobiosensors with enough selectivity and sensitivity for the detection of contaminants in real samples.

## 5. Integration with Microfluidic Systems

In recent years, the development of microfluidic devices has revolutionized analytical chemistry, enabling high-resolution separations, the synthesis of monodisperse colloidal systems, and the rapid, cost-effective detection of multiple analytes [190,191]. Using this technology, small volumes of fluids can be controlled and analyzed at the scale of micrometers. Their high surface-to-volume ratio plays a critical role in enhancing mass transfer and reaction kinetics, and optimizing chemical and biological processes for sensitive and efficient detection [192]. This characteristic is particularly relevant in water quality monitoring, where microfluidic platforms facilitate the real-time detection of contaminants with minimal sample volumes, increased precision, and automated data acquisition [193].

The foundation for these advancements can be traced back to pioneering works in microfluidics, such as the study by Wang et al. [194]. Their work demonstrated the feasibility of fully disposable microfluidic platforms with high analytical performance, sensitivity, and reproducibility, paving the way for the practical implementation of “lab-on-a-chip” technologies in clinical and environmental analysis. Along with other works from this era, it laid the groundwork for the development of this transformative technology.

**Figure 7:**
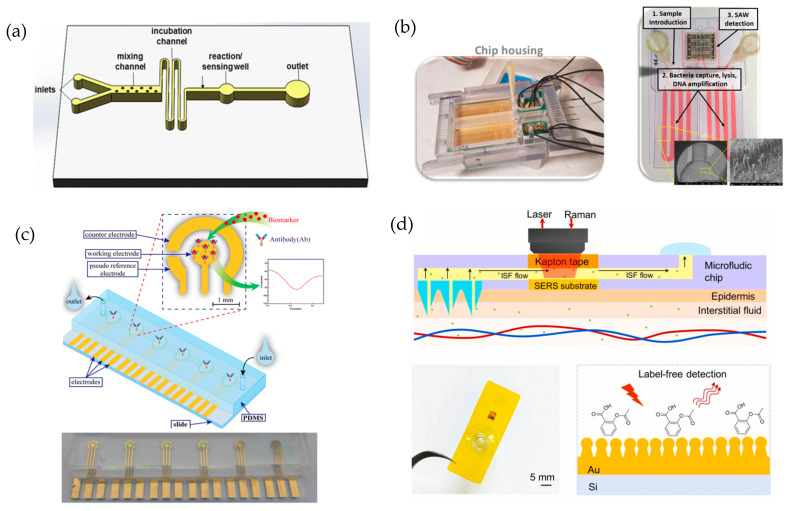
(**a**) General representation of a microfluidic device, illustrating key components for fluid mixing, incubation, reaction, and sensing, which are essential for analytical applications. Reprinted from Weng et al. [200], copyright 2015, Springer Nature. (**b**–**d**) Different configurations of microfluidic devices integrated with biosensors, depending on the detection method used: (**b**) illustration of SAW-based detection, reproduced from Tsougeni et al. [201], copyright 2020, Elsevier; (**c**) representation of a biosensor utilizing electrochemical measurements, adapted from Yao et al. [202], copyright 2015, Springer Nature; (**d**) biosensor based on SERS spectroscopy, adapted from Xiao et al. [203], copyright 2020, Elsevier.

Microfluidic systems significantly improve biosensor performance by addressing key challenges in water analysis, including low contaminant concentrations, complex sample matrices, and the need for rapid on-site measurements. The integration of miniaturized microchannels optimizes fluid dynamics, ensuring enhanced mixing, controlled reaction times, and efficient analyte transport to detection zones [195]. For this reason, microfluidic devices consist of several interconnected sections (Figure 7a). The process begins at the inlets, where fluids, such as reagents or biological samples, are introduced. These fluids then move through a mixing channel designed to combine liquids homogeneously [196]. Next, they pass through the incubation channel, which extends the residence time to complete chemical or biological reactions [197]. Finally, in the detection chamber, integrated sensors analyze specific parameters, such as molecular concentrations, before the processed fluid exits via the outlet [198]. These configurations can vary depending on the design, with straight, Y-shaped, or T-shaped flow geometries [196,197,198,199].

By leveraging precisely controlled fluid dynamics, microfluidic biosensors for water quality monitoring achieve enhanced detection sensitivity, rapid response times, and cost-effective operation. The ability to integrate multi-analyte detection on a single chip, coupled with automated sample preparation and analysis, further demonstrates the transformative potential of microfluidics in environmental monitoring.

### 5.1. Materials and Fabrication Techniques

One of the most critical aspects in the development of microfluidic devices is material selection, as it directly influences biosensor biocompatibility, performance, reproducibility, and durability. Commonly used materials include rigid polymers, polydimethylsiloxane (PDMS), glass, silicon, paper, and 3D-printed composites [199]. PDMS is highly valued for its flexibility, optical transparency, and compatibility with micromolding techniques, facilitating the creation of high-resolution microchannels. However, its tendency to absorb hydrophobic compounds and high gas permeability may affect assay reproducibility, particularly in quantitative detection biosensors [204].

On the other hand, thermoplastics such as polymethyl methacrylate (PMMA) and polycarbonate (PC) offer greater mechanical stability and lower biomolecule absorption, making them more suitable for precise analytical applications. PMMA stands out for its transparency, lightness, and ease of integration with optical methods, while PC is highly impact-resistant, making it an ideal choice for portable environmental monitoring devices [205]. Additionally, materials such as polystyrene (PS) and cyclic olefin copolymer (COC) are attractive options due to their low cost, ease of molding, and chemical resistance, making them ideal for disposable biosensors and high-throughput analysis [199,205].

An emerging approach in microfluidics is the use of paper-based microfluidic devices (μPADs), which enable passive fluid flow through capillary action, eliminating the need for external pumps [206]. These devices have proven particularly useful for point-of-care detection, such as rapid pathogen identification and environmental monitoring. The integration of electrochemical and colorimetric biosensors into μPADs has led to the development of low-cost, highly sensitive disposable devices suitable for field applications [206,207].

Beyond material selection, fabrication techniques play a key role in the precision, reproducibility, and scalability of microfluidic biosensors. Soft lithography remains one of the most used techniques for PDMS devices, allowing for the high-resolution fabrication of three-dimensional microchannels. However, the low mechanical strength of PDMS and its challenges in large-scale production have driven the development of alternative techniques such as injection molding and micromachining, especially for PMMA, PC, and COC-based devices, which offer greater robustness and reproducibility in mass production [208,209].

Laser-based techniques, such as engraving and ablation, enable the direct fabrication of microchannels on thermoplastics, reducing costs and eliminating the need for molds while maintaining high precision [210]. Laser direct writing uses photopolymerization to create fluidic patterns with minimal contamination, while CO₂ laser micromachining optimizes surface quality and dimensional accuracy in materials like PMMA [211]. Additionally, the use of chemical processes such as inkjet printing facilitates the high-resolution deposition of biomolecules, facilitating the fabrication of biosensors on paper and flexible surfaces. However, reactant adhesion can present a challenge in these devices, requiring the development of improved biomolecule fixation techniques [204].

Advancements in material selection and fabrication techniques have significantly driven the development of high-performance microfluidic biosensors, with applications in pathogen detection, environmental monitoring, and clinical diagnostics. The combination of materials such as PMMA and PDMS with optimized manufacturing processes, such as injection molding and laser engraving, has led to the creation of more efficient and reproducible devices. However, challenges remain, such as long-term mechanical stability, adhesion optimization in biosensors, and reducing production costs [212]. As research in this field progresses, the integration of new manufacturing strategies and the use of innovative materials such as polyimide and stainless steel could enhance the functionality and scalability of these devices in real-world applications [213].

### 5.2. Applications of Microfluidics: From Lab to Real-World Samples

Beyond manufacturing, microfluidic devices significantly enhance biosensor performance in water quality monitoring by enabling precise fluid control, reducing sample volume, and improving reaction efficiency. These advantages are crucial for real-time contaminant detection, where achieving high sensitivity with minimal sample and reagent consumption is essential. This is particularly evident in various advanced analytical techniques, as illustrated by SWA (Figure 7b), electrochemistry (Figure 7c), and SERS (Figure 7d).

Multiple studies were conducted in synthetic water matrices, ensuring controlled laboratory results. One example is the study by Nissen et al. [214], who integrated water-filled antiresonant hollow fibers into a microfluidic system for UV spectroscopy. Their approach significantly enhances (100-fold longer) light–matter interactions, improving the detection of pharmaceutical residues like sulfamethoxazole and sodium salicylate at 0.1 μM and 0.4 μM concentrations, respectively. By reducing the sample volume by 99.9%, this technique exemplifies how microfluidic integration improves efficiency in environmental analysis, making it ideal for water quality monitoring. Moreover, Burtsev et al. [215] developed a SERS-based system to trace ibuprofen in water, achieving a detection limit of 10^−2^ μM. By combining an optimized mixer with functionalized gold nanoparticles, the system effectively captures the analyte without requiring prior sample preparation, offering high sensitivity even in complex wastewater matrices.

In the field of pathogen detection, microfluidic biosensors enable rapid, automated, and highly specific quantification, which is particularly valuable in food safety and environmental applications. A clear example of this is the work by Tsougeni et al. [201], who developed a lab-on-a-chip system that integrates immunological capture, chemical lysis, isothermal amplification, and acoustic detection for the identification of pathogen such as *Salmonella*, *Listeria*, and *E. coli*. By optimizing microfluidic flow control, the system enhanced reaction kinetics, reducing the analysis time by five-fold compared to conventional methods, achieving a detection limit of 1–5 CFU per 25 mL within 4.5 h. Additionally, Man et al. [216] presented a portable colorimetric biosensor based on a microchip for detecting *Salmonella* in fresh produce. Using functionalized polystyrene nanoparticles and an innovative valve, this device achieves detection within 45 min at a limit of 60 CFU/mL. The results are captured via a mobile app, offering accessibility and cost-effectiveness. Similarly, Jiang et al. [217] proposed a dual RCA-based microfluidic platform to detect *E. coli* O157:H7. By enhancing sensitivity with repeated aptamers and signal amplification, the system achieves detection limits of 80 cells/mL, amplifying signals up to 250-fold. For the same pathogen, Zheng et al. [218] introduced a colorimetric biosensor combining Au nanoparticles with a microfluidic chip to the analysis of chicken samples. This system combines portability and sensitivity, achieving detection within 50 min at a limit of 50 CFU/mL, making it ideal for field-based food safety applications.

Although most studies focus on the detection of pathogens, there are also applications in the environmental field, as demonstrated by the work of Ben Messaoud et al. [88]. As highlighted in Section 2.2, this study introduces an innovative, portable, label-free, and ultrasensitive electrochemical immunosensor designed for the detection of *A. salmonicida* in seawater. Also, the main innovation of their work lies in the use of a fluidic-integrated electrochemical-cell chip, which features independent chambers housing three electrochemical cells. This not only allows for a compact and portable design but also enhances the capability for simultaneous and reproducible analysis. Furthermore, the sensor offers a wide linear working range, spanning from 1 to 10^7^ CFU/mL, and an extremely low LOD of 1 CFU/mL.

Nevertheless, integrating microfluidics with biosensors has enabled the precise detection of contaminants in real water samples, facilitating environmental monitoring and real-time contaminant identification [219]. A notable example is the study by Doostmohammadi et al. [220], who developed a portable microfluidic biosensor based on cell-imprinted polymers (CIPs) for detecting bacteria in pond water. The device, integrated into a 3D-printed platform, utilized magnetically functionalized CIP microparticles within a magnetophoretic microfluidic system. Tests performed in pond water revealed an *E. coli* concentration of 2 × 10^6^ CFU/mL, closely matching results obtained in a central laboratory (2.33 × 10^6^ CFU/mL). However, the non-specific bacteria presence, including *Salmonella* and *Sarcina*, compromised the sensor’s selectivity, highlighting the need for optimization in more complex aquatic matrices.

In a similar approach, Akhtarian et al. [221] developed a microfluidic biosensor based on EIS, incorporating CIPs for *E. coli* detection in water. This sensor featured stainless steel microthreads functionalized with CIPs within a PDMS microchannel, enabling rapid and cost-effective detection in environmental samples. When tested in real water, the sensor successfully differentiated *E. coli* concentrations ranging from 10^2^ to 10^7^ CFU/mL, with a detection limit of 2 × 10^2^ CFU/mL. However, ensuring sensor stability under varying environmental conditions remains a challenge, requiring further optimization to enhance robustness in untreated water matrices.

Further advancing this technology, Akhtarian et al. [222] also designed a conductometric sensor utilizing microthreads coated with CIPs for bacterial detection in real water samples. The sensor exhibited a quantifiable response between 10^4^ and 10^7^ CFU/mL of *E. coli*, with a sensitivity of 7.35 µS per CFU/mL. Tests conducted in pond water revealed a decrease in electrical resistance following bacterial capture, enabling effective quantification. Notably, in this case, the presence of other bacteria such as *Sarcina* and *Listeria* did not significantly impact sensor specificity, suggesting that CIP technology could be a promising approach for the selective detection of pathogens in water.

However, microfluidics is not only used for pathogen quantification but also plays a crucial role in the detection of pharmaceutical contaminants and heavy metals, as demonstrated by Hidi et al. [199] and He et al. [223], respectively. Hidi et al. [199] introduced a lab-on-a-chip platform integrated with SERS for ciprofloxacin detection in river water. By optimizing the pH to 11, the chemical affinity between ciprofloxacin and silver nanoparticles was enhanced, significantly improving the SERS signal. The system achieved a detection range of 0.7–10 μM with a detection limit below 0.74 μM, which corresponds to the minimum inhibitory concentration for relevant bacterial strains. Similarly, He et al. [223] developed a microfluidic biosensor based on SERS for the ultrasensitive detection of uranyl ions in drinking and river water. This sensor utilized ZnO-Ag hybrids modified with aptamers, allowing highly specific detection with a detection limit as low as 7.2 × 10⁻^7^ μM, five orders of magnitude below the maximum level permitted by the US Environmental Protection Agency. When tested in river and tap water, the sensor demonstrated effective analyte recovery, although interferences from other metal ions, such as Pb^2^⁺, Hg^2^⁺, and Cu^2^⁺, were detected, potentially compromising measurement accuracy in complex environmental samples.

This challenge in selective detection was also observed in the studies by Doostmohammadi et al. and Akhtarian et al. [220,222], where the presence of non-specific bacteria such as *Salmonella, Sarcina*, and *Listeria* impacted sensor specificity. These findings underscore the importance of optimizing selectivity in real water matrices to ensure precise measurements across varying environmental conditions.

The relevance of microfluidics in the quantification of contaminants in real water samples is highlighted and demonstrated the impact of different detection strategies on biosensor precision and sensitivity. The presence of interferences in environmental samples remains a major challenge, emphasizing the need for more selective and robust technologies for field applications. As these devices continue to evolve, their integration with artificial intelligence and automated analysis could significantly enhance performance in environmental monitoring and water safety applications.

### 5.3. Integration of Artificial Intelligence in Microfluidics

Significant advancements in artificial intelligence (AI) have enabled its integration into various technological and scientific fields, including water quality monitoring. AI branch machine learning (ML) and deep learning (DL) have arisen as ideal tools for the design of microfluidic systems based on enhancing the automation, speed, and precision of the related tasks [224]. ML aims to create models from sample data in terms of task and performance metrics, and DL is an ML approach based on complex neural networks to extract the inherent patterns and features from enormous databases.

The incorporation of AI to microfluidic devices holds great potential for advancing the next-generation monitoring systems to attain time-efficient setups and easy operation. This has led to the development of “intelligent microfluidics”, where ML algorithms optimize the design, operation, and analysis of microfluidic biosensors used for environmental applications. By leveraging AI, traditional limitations such as manual calibration, experimental variability, and human intervention in water analysis have been mitigated, enhancing detection speed, accuracy, and automation [225,226,227].

AI-driven microfluidic biosensors have demonstrated remarkable improvements in contaminant detection, particularly in the real-time analysis of heavy metals, pesticides, pharmaceutical residues, and microbial pathogens in surface water, drinking water, and industrial wastewater. A key example is flow control within microchannels, where neural networks dynamically adjust parameters to maintain stability in droplet-based sensing platforms, ensuring optimal reagent mixing and sample processing [225,228]. In biosensing applications, computer vision algorithms facilitate the automated detection of pollutants, including lead (Pb^2^⁺), mercury (Hg^2^⁺), and nitrates, through AI-enhanced optical and electrochemical sensors, significantly reducing the detection time and increasing precision. The optimization of microfluidic chip design through AI has enabled the development of more efficient and scalable water analysis systems, minimizing reagent consumption and improving assay reproducibility. Predictive models are now capable of estimating flow dynamics, reaction efficiency, and sensor performance under varying environmental conditions, ensuring robust operation in complex water matrices. Additionally, DL-based algorithms have been employed for the automated classification of microbial contaminants, achieving an accuracy of over 96% in pathogen identification, crucial for preventing waterborne diseases [228].

Another key advancement is the development of portable AI-powered microfluidic devices for on-site water testing, reducing the dependency on centralized laboratories. These systems leverage real-time data processing to analyze water samples instantly, providing rapid alerts for contamination events. For instance, AI-integrated microfluidic sensors have been deployed for the early detection of harmful algal blooms, monitoring toxin concentrations and predicting outbreaks before they escalate. Similarly, AI-enhanced electrochemical biosensors detect antibiotics and endocrine-disrupting chemicals in wastewater, enabling faster decision making in water treatment facilities [224].

Despite these advances, challenges persist, particularly the requirement for extensive datasets to train AI models, ensuring compatibility across different platforms, and addressing sensor fouling and biofouling issues in real-world water samples. Additionally, material selection plays a crucial role in improving microfluidic biosensor performance, as biocompatibility, chemical resistance, and sensor stability must be considered for long-term environmental applications. While PDMS, PMMA, and thermoplastic-based biosensors have shown promise in lab conditions, further research is needed to enhance their durability and adaptability for field applications [225,229].

In summary, these advances in microfluidic devices enhance sensitivity, reduce analysis time, and enable portable, cost-effective solutions for environmental monitoring. However, for widespread adoption, it is essential to validate these systems using real water samples, ensuring their reliability in detecting trace contaminants such as heavy metals, pesticides, and pharmaceutical pollutants. With ongoing improvements in material engineering, AI integration, and reproducibility, microfluidic biosensors are poised to become key tools in water quality monitoring and beyond.

## 6. New Trend in Biosensors: Molecularly Imprinted Polymers

**Figure 8:**
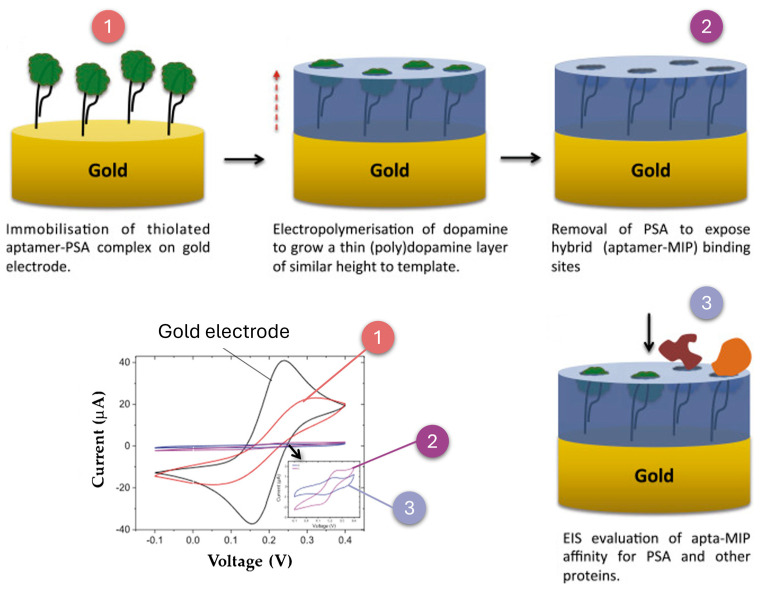
Schematic representation of the fabrication process of an Apta-MIP electrode. The aptamer is immobilized on the electrode surface, followed by dopamine electropolymerization, forming a molecularly imprinted polymer (MIP). After washing to remove PSA, aptamer-lined imprinted cavities remain, enabling selective recognition, a process that can be monitored using CV. This is an adaptation of Jolly et al. [237], copyright 2016, Elsevier.

Molecularly imprinted polymers (MIPs) are synthetic polymers designed to exhibit highly specific recognition capabilities for a target molecule. These materials are produced by polymerizing functional monomers around a template molecule, which is then removed to leave behind cavities complementary in shape, size, and chemical properties to the template [230]. Figure 8 illustrates the formation process combining MIPs with aptamers (Apta-MIPs), highlighting the key steps in their fabrication. This innovative approach has been increasingly adopted in recent years to address challenges in the detection of contaminants such as pharmaceuticals, pesticides, and pathogens. MIPs have emerged as a powerful alternative to traditional detection methods, offering advantages like stability, reusability, and high selectivity even in complex sample matrices [231,232].

Several studies highlight the growing relevance of MIPs in environmental, food safety, and medical applications. Rocha et al. [233] demonstrated the effectiveness of an electrochemical MIP-based sensor for the selective detection of tetrodotoxin in seafood samples, achieving low detection limits and excellent reproducibility in complex matrices. Similarly, Wang et al. [234] showcased the use of MIPs for the selective detection of tetracycline residues in environmental and biological samples, emphasizing their potential to address the persistence of antibiotic contaminants in ecosystems. In addition, Minhas et al. [235] demonstrated the utility of MIPs in the efficient extraction of bioactive compounds, such as methyl gallate from plant extracts, improving selectivity and streamlining the extraction process for complex matrices.

Electrochemical sensors based on MIPs further illustrate their versatility. Shalapy et al. [236] developed a novel MIP sensor for detecting indigo carmine dye in food samples, achieving ultra-trace sensitivity and exceptional selectivity. By integrating molecular imprinting with advanced sensing platforms, this work highlighted the potential of MIPs for rapid, in situ detection, overcoming the limitations of traditional chromatographic and spectrophotometric methods.

Agar et al. [238] introduced an innovative hybrid approach using Apta-MIPs, leveraging the robust properties of MIPs with the enhanced specificity of aptamers. This dual recognition system enabled the multiplexed detection of bacteria such as *E. coli* and *S. aureus* in water, achieving high sensitivity and selectivity with detection limits as low as 2 CFU/mL for *E. coli*. Apta-MIPs not only enhance detection capabilities but also broaden the scope of applications, making them a promising tool for point-of-care diagnostics and water quality monitoring.

In another innovative application, MIPs were combined with fluorescence-based nanomaterials, such as Si-doped biomass-derived carbon dots, to create highly selective nanosensors. Wang et al. [234] demonstrated this by designing a MIP-based nanosensor for the detection of rutin, a bioactive flavonoid, in herbal medicine. This combination leverages the high specificity of MIPs and the optical properties of carbon dots, enabling rapid and accurate detection in complex biological matrices.

Additionally, Surface Molecular Imprinting Technology (SMIP) has emerged as a promising evolution of MIP technology. Unlike traditional bulk imprinting methods, SMIP creates imprinted cavities on the surface of substrates, significantly improving the accessibility and binding efficiency of target molecules. Li et al. [239] highlighted the integration of SMIPs with SERS technology, which combines the selective preconcentration capabilities of SMIPs with the signal amplification of SERS. This hybrid system provides a powerful tool for detecting trace contaminants in complex matrices, offering a unified approach for sample pre-treatment and analysis, especially in fields like environmental monitoring and food safety.

These examples illustrate how MIPs, in their various forms and combinations with other technologies, have transformed contaminant detection. The continued integration of MIPs with cutting-edge techniques such as aptamers, fluorescence-based nanomaterials, and SMIP-SERS platforms is expected to further expand their applications, addressing global challenges in public health, environmental safety, and sustainable development.

## 7. Challenges and Future Prospects

Throughout this review, the potential of biosensors for environmental monitoring has been highlighted. However, various challenges must be addressed to fully exploit their capabilities. These challenges include issues related to sensitivity, stability, standardization, and the exploration of new technological directions.

One of the main challenges lies in achieving the required sensitivity and specificity to detect trace contaminants in complex environments, such as wastewater. Like other analytical techniques, biosensors often face interferences from organic matter, chemical pollutants, and microbial diversity, which can compromise their accuracy and hinder their application in real-world samples. As discussed in Section 2.1 and Section 2.2, strategies such as signal amplification and the integration of nanomaterials could further enhance detection capabilities by improving specificity and reducing noise. Another significant challenge for biosensors is ensuring their durability and reproducibility, which remain major obstacles to their widespread adoption. Variable environmental conditions, including changes in pH, temperature, and salinity, can degrade biosensor components, leading to inconsistent results. Proposed strategies to mitigate these effects include the use of robust protective materials and coatings, the application of synthetic biology to develop more stable bioreceptors, and the implementation of real-time calibration and self-diagnostic systems to ensure consistent performance.

Future research should prioritize exploring innovative strategies to enhance the functionality and applicability of biosensors. Achieving seamless integration with existing monitoring systems is critical to improving their usability and efficiency in large-scale applications. The development of multiplexed biosensors capable of detecting multiple contaminants simultaneously could significantly increase operational efficiency and broaden their applicability. Furthermore, advancing battery-free biosensor technologies—powered by energy harvesting mechanisms such as piezoelectric or photovoltaic systems—would enable sustainable, long-term deployment in remote or resource-limited areas. Expanding wearable and portable biosensor technologies will also facilitate rapid, on-site monitoring, significantly decreasing the reliance on centralized laboratories. By addressing these challenges and focusing on these future directions, biosensors can reach their full potential as indispensable tools for environmental monitoring.

In summary, overcoming the challenges of sensitivity, stability, seamless integration, expanded functionality, and battery-free operation is crucial for the widespread adoption of biosensors in environmental monitoring. Sensitivity and stability are key to ensuring reliable detection in diverse conditions, while seamless integration and multifunctionality will enhance their adaptability and real-time usability. Battery-free operation further enables sustainable and remote deployments. Additionally, establishing clear and consistent guidelines for standardization is essential to ensure comparability, reproducibility, and regulatory acceptance. By leveraging these advancements, biosensors can become indispensable tools for safeguarding water quality and environmental health.

## 8. Conclusions

For more than three decades, biosensors have represented a transformative technology with multiple applications spanning medicine, food safety, and environmental monitoring. This review underscores their potential as critical tools for water quality assessment, particularly for detecting emerging contaminants (ECs) and pathogens. By classifying biosensors based on their bioreceptor and transducer, the discussion highlights how these devices are being tailored to address the challenges of monitoring pollutants in complex aquatic environments.

Recent innovations such as SELEX, SAMs, and MIPs have significantly enhanced the sensitivity and selectivity of biosensors. SELEX enables the development of highly specific aptamers, which serve as efficient recognition elements for diverse analytes, while SAMs provide functionalized surfaces that ensure stable biomolecule immobilization and minimize non-specific interactions. Additionally, microfluidic systems have revolutionized biosensor integration by improving detection speed, enabling real-time analysis, and reducing the sample volumes required for testing.

Despite these advancements, challenges such as achieving the required sensitivity in complex wastewater environments, ensuring long-term stability, and addressing regulatory barriers remain. Future research should focus on the development of multiplexed biosensors, the incorporation of artificial intelligence for advanced data analysis, and the creation of sustainable and portable devices.

In conclusion, biosensors are positioned to become indispensable tools for safeguarding public health and protecting environmental integrity. Continued innovation and collaboration across disciplines will be critical to overcoming existing challenges and unlocking their full potential.

## Figures and Tables

**Table 2:** Classification of optic biosensors for pathogens and emerging pollutants in terms of target, optical signal, biorecognition element, and LOD.

Target	Optical Signal	Biorecognition Element	LOD	Reference
**Pathogens**				
*S. aureus*	SERS	*S. aureus* aptamer	16 CFU/mL	[155]
**Pharmaceuticals and PCPs**				
Diclofenac (DCF)	SPR	Anti-DCF	1.06 × 10^−2^ µM	[156]
DCF	QCM with dissipation (QCM-D) and localized surface plasmon resonance (LSPR).	Anti-DCF	9.49 × 10^−3^ µM	[157]
Azithromycin	Ratiometric fluorescence	Azithromycin aptamer	9.78 × 10^−3^ µM	[158]
Amoxicilin	UV-Vis	DNA-Aptamer	6.70 × 10^−5^ µM	[159]
**Pesticides and agrochemicals**				
Imidacloprid Pyraclostrobin	SERS	Antibody	3.36 × 10^−5^ µM 2.51 × 10^−4^ µM	[160]
Dichlorvos	UV-Vis	AChE enzyme	0.65 μM	[161]
**Perfluorinated compounds**				
PFOA PFOS	Ratiometric fluorescence	Defluorinase enzyme	2.42 × 10^−5^ µM 2.00 × 10^−5^ µM	[162]
**Antimicrobial and disinfectants**				
Chlorophene	SPR	Laccase enzyme	1.51 µM	[163]
Triclosan	Raman	BSA-protein	0.05 µM	[154]
**Nanoplastics**				
Polymethyl Methacrylate	SPR	Estrogen receptor	1.02 × 10^−3^ µg/mL	[164]
**Mycotoxins**				
Aflatoxina B1	Raman	Antibody	2.85 × 10^−5^ µM	[160]
